# Real-time PCR assays for detection and quantification of early *P. falciparum* gametocyte stages

**DOI:** 10.1038/s41598-021-97456-4

**Published:** 2021-09-27

**Authors:** Amal A. H. Gadalla, Giulia Siciliano, Ryan Farid, Pietro Alano, Lisa Ranford-Cartwright, James S. McCarthy, Joanne Thompson, Hamza A Babiker

**Affiliations:** 1 College of Medicine and Health Sciences, Sultan Qaboos University, Muscat, Oman; 2 Division of Population Medicine, School of Medicine, College of Biomedical Sciences, Cardiff University, Cardiff, UK; 3 Dipartimento di Malattie Infettive, Istituto Superiore di Sanità, Rome, Italy; 4 QIMR Berghofer Medical Research Institute and University of Queensland, Brisbane, Australia; 5 Institute of Biodiversity, Animal Health and Comparative Medicine, College of Medical, Veterinary and Life Sciences, University of Glasgow, Glasgow, UK; 6 Institute of Immunology and Infection Research, School of Biological Sciences, Ashworth Laboratories, University of Edinburgh, Edinburgh, UK

**Keywords:** Infectious-disease diagnostics, Microbial genetics, Parasitology

## Abstract

The use of quantitative qRT-PCR assays for detection and quantification of late gametocyte stages has revealed the high transmission capacity of the human malaria parasite, *Plasmodium falciparum*. To understand how the parasite adjusts its transmission in response to in-host environmental conditions including antimalarials requires simultaneous quantification of early and late gametocytes. Here, we describe qRT-PCR assays that specifically detect and quantify early-stage *P. falciparum* gametocytes. The assays are based on expression of known early and late gametocyte genes and were developed using purified stage II and stage V gametocytes and tested in natural and controlled human infections. Genes *pfpeg4* and *pfg27* are specifically expressed at significant levels in early gametocytes with a limit of quantification of 190 and 390 gametocytes/mL, respectively. In infected volunteers, transcripts of *pfpeg4* and *pfg27* were detected shortly after the onset of blood stage infection. In natural infections, both early (*pfpeg4*/*pfg27*) and late gametocyte transcripts *(pfs25*) were detected in 71.2% of individuals, only early gametocyte transcripts in 12.6%, and only late gametocyte transcripts in 15.2%. The *pfpeg4*/*pfg27* qRT-PCR assays are sensitive and specific for quantification of circulating sexually committed ring stages/early gametocytes and can be used to increase our understanding of epidemiological processes that modulate *P. falciparum* transmission.

## Introduction

The malaria parasite *Plasmodium falciparum* continues to be a major cause of human disease despite mounting control measures. Central to this success is its ability to undergo sexual development and efficient transmission between human hosts through its mosquito vector. In *P. falciparum*, sexual development occurs when asexual blood stage parasites exit the proliferative cycle and develop into schizonts containing merozoites committed to sexual differentiation ^1^. These merozoites invade erythrocytes and differentiate into the sexual forms, gametocytes, over a period of approximately 10 days, passing through five morphologically distinct stages (I to V)^2^. A second direct path to commitment to gametocytogenesis may also occur shortly after invasion of the erythrocyte by an uncommitted merozoite^3^*.* While sexually committed ring stages/very early stage I gametocytes and late stage V gametocytes are present in the peripheral circulation, immature gametocytes (stage II-IV) withdraw from peripheral circulation and sequester in tissue microvasculature^4–7^.

Stage I gametocytes are morphologically indistinguishable from pigmented asexual trophozoites, and thus cannot be identified in peripheral blood by microscopic examination. Transcriptome analyses have led to the identification of transcripts upregulated before morphological differentiation into stage II gametocytes. These include *pfmdv-1 (pfpeg3), pfgdv1*, *pfge1*-*3*^8–10^ and *pfpeg4*^11^*,* which encodes the membrane protein ETRAMP 10.3^8,12,13^. Further analyses of the transcriptomes of *P. falciparum* isolates have demonstrated that transcripts of *pfge1*, *pfge2* and *pfg27* cluster away from those of late stage-specific genes *pfs25*, *pfs28* and *pfs47,* suggesting that they are expressed exclusively in early stage gametocytes^10^. In addition, Tiburcio et al.^14^ described PfGEXP5 as the earliest gametocyte-specific protein, expressed 14 h post-invasion. This is consistent with the detection of the *pfgexp5* transcript in blood samples taken at early time points following experimental infection of volunteers with *P. falciparum*^15^.

The development of RT-PCR assays based on late sexual stage-specific mRNA transcripts has enabled the conduct of epidemiological surveys to study the transmission potential of *P. falciparum* in the face of continued control efforts^16–19^. These surveys have identified a range of epidemiological correlates that modulate transmission, including drug treatment, anaemia, mixed species infection and multiplicity of genotypes^20–23^. These epidemiological findings are consistent with the predictions of the evolutionary hypothesis that the interplay between asexual replication and transmission is altered by a changing in-host environment^24,25^. However, to disentangle the effect of these factors on transmission success, there is a need for assays that can detect and quantify very early gametocytes close to the point of commitment.

Here, we describe sensitive qRT-PCR assays for quantification of early stage gametocytes, that complement existing late-stage assays, to enable understanding of epidemiological factors that drive *P. falciparum* transmission success*,* and robust assessment of control strategies targeting sexual stages. The assays were validated using in vitro purified early and late *P. falciparum* gametocytes and their robustness was tested using samples from natural infections and experimentally-infected volunteers.

## Methods

### Asexual and purified stage II and V gametocyte samples

*Plasmodium falciparum* 3D7^26^ was obtained from The European Malaria Reagent Repository (http://www.malariaresearch.eu). Asexual and sexual culture stages were cultivated following the protocol of Trager and Jensen^26^ with minor modifications. Briefly, parasites were maintained in human type O-positive RBCs at 5% haematocrit (Hct) in RPMI 1640 medium supplemented with 25 mM HEPES (Sigma-Aldrich, UK), 50 µg/ml hypoxanthine and with the addition of 10% (v/v) naturally-clotted heat-inactivated 0 + human serum (Interstate Blood Bank, Inc.). The cultures were maintained at 37 °C in a standard gas mixture consisting of 3% O_2_, 5% CO_2_ and 92% N_2_. To obtain synchronous gametocytes, an asexual culture (0.5–1% starting parasitaemia) was grown without further dilution as described^27^. Induced gametocyte cultures were supplemented with 50 mM N-acetylglucosamine (NAG; Sigma-Aldrich, UK) to clear residual asexual parasites and obtain a virtually pure gametocyte culture^28^. To obtain the stage II gametocyte sample, two days after NAG treatment gametocytes were inspected on Giemsa-stained smears to assess maturation, and purified by centrifugation through a discontinuous Percoll gradient, as described in Silvestrini et al.^29^. Gametocytes were examined in a counting chamber for quantification and control of contamination. Stage IV and V gametocytes were not detectable. A small fraction of residual unhealthy/dead trophozoites and uninfected RBCs was detectable. To obtain the stage V gametocyte sample, gametocytes were induced as described before^27^, and 12 days after NAG treatment gametocytes were purified by MACS Separation Columns CS (Miltenyi Biotec, Germany)^30^ and examined as above. Immature sexual stages and asexual stages could not be detected. In both cases the enriched parasite preparations were pelleted (5 × 10^5^ gametocytes/pellet) and frozen in liquid nitrogen. Samples containing mixed-stage asexual parasites, purified stage II gametocytes, and purified stage V gametocytes were used to examine the stage specificity of the candidate early gametocyte genes and to establish the mathematical relationship between transcript numbers of the candidate genes and gametocyte counts (more details are given in Material and Methods section "qRT-PCR assays for early and late gametocytes").

### Natural *P. falciparum* infections

A total of 250 samples were examined from an existing collection, stored at − 80 °C as packed RBCs. The samples were obtained from patients with uncomplicated *P. falciparum* malaria from Asar village, eastern Sudan. All participants gave informed consent to participate in the study and ethical approval was obtained from the National Health Research Ethics Committee, Ministry of Heath, Khartoum, Sudan^31^. All experiments were performed in accordance with the relevant guidelines and regulations.

These samples were employed in the current study to investigate whether the expression of early gametocyte markers is: (1) detectable in natural infections; (2) detectable specifically in a subset of samples that were *pfs16* positive (early and late gametocyte marker) and *pfs25* negative (late gametocyte marker); (3) associated with total parasitaemia.

### Experimentally infected human volunteers

Samples from experimentally-infected human volunteers, who had participated in a previously published clinical trial^15,32^, were utilized to study the stage-specificity of the gametocyte markers in vivo. Ethical approval of the study was obtained from QIMR Berghofer Human Research Ethics Committee and all participants gave informed, written consent to participate in the study^32^. All experiments used for analyses of these samples were performed in accordance with the relevant guidelines and regulations.

The volunteer cohort has been previously described. In brief, it comprised malaria-naïve, healthy males and non-pregnant females, aged 18–50 years. Infections were induced using approximately 1800 viable *P. falciparum*–infected human erythrocytes of clone 3D7. Volunteers were then treated with 480 mg of piperaquine (which affects only asexual stages) on Day 8 (D08) post-infection, when parasitaemia reached a predetermined threshold of > 1,000 parasites/mL. In the current study, samples were examined from two of these volunteers who showed a relatively high post-treatment parasitaemia, and high levels of *pfs25* expression at or beyond D11 post-infection^15^. Ten time points were selected for each volunteer, between D07 to D09 post-infection. This represents a suitable window for interrogation for early gametocytes, as parasitaemia started to increase at D07, with limited possibility for the presence of late gametocytes in the initial inoculum as discussed^15^.

### Total parasite density quantification

Parasite density was quantified as parasites/mL of blood using absolute quantification of the *18S rRNA* gene by qPCR assay^33,34^. *18S rRNA* copy numbers were converted to parasite numbers using a calibration curve from *P. falciparum* clone 3D7 parasite DNA with a range of 0.14 to 138,938 parasites/μL of DNA^35^. The *18S rRNA* qPCR amplification efficiency was 97.7% (se 0.01%). Quantification was carried out in duplicate with maximal permitted standard deviation of 0.35. Quantification of parasite density in the volunteers’ samples has been described elsewhere^15^.

### DNA, RNA extraction, purification and cDNA preparation

For samples from naturally-infected patients, DNA and RNA were extracted from 100 µL and 50 µL of packed RBCs, using the QIAamp DNA mini kit (Qiagen, UK) and SV Total RNA Isolation System (Promega, UK) respectively. For RNA purification, all samples were treated with a unified concentration of 1 unit of RQ1 RNase-Free DNase (Promega, UK) per 8 µL of RNA sample to remove any genomic DNA (gDNA) carryover. Then, purified RNA samples were checked for gDNA by*18S rRNA* qPCR assays. Pure RNA samples were converted to cDNA using the High-Capacity cDNA Reverse Transcription Kit with random primers (Thermo Fisher, UK). For the volunteer cohort, RNA was extracted from 800 µL of whole blood and cDNA was prepared as described^15^.

### qRT-PCR assays for early and late gametocytes

#### Oligonucleotides

Full gene names and accession numbers are provided in supplementary Table S1. qRT-PCR primers and TaqMan dual-labelled probes were designed within exon sequences, avoiding polymorphic regions to ensure reproducibility of the experiment for field isolates (Table S2). BLAST alignments^36,37^ against sequences available in PlasmoDB.org^37^ showed that the primers and probe sets had 100% identity with *P. falciparum* target genes, and no high identity alignment with other human *Plasmodium* species or other *P. falciparum* genes. This was further confirmed by the absence of amplification when primers and probes were used in PCR assays using *Plasmodium vivax*, *Plasmodium malariae* and *Plasmodium ovale* gDNA. Probe design for *pfgexp5* was described previously by Farid et al.^15^.

#### Optimization

A Taqman assay was designed and optimised for each of the putative early gametocyte-specific genes (*pfpeg4*, *pfg27*, *pfge1*, *pfge3* and *pfgexp5*), for one late gametocyte-specific gene (*pfs25*), for one pan-gametocyte marker (*pfs16*), and for one reference gene (*pf40S*). Primer concentrations for the optimised qRT-PCR reactions were 0.3 µM (*pfpeg4*, *pfg27*, *pfge1* and *pfs25*), 0.6 µM (*pfgexp5*), and 1.2 µM (*pfge3*). Probe concentrations used were 0.1 µM in the *pfpeg4*, *pfge1*, *pfge3*, *pfgexp5* and *pfs25* qRT-PCR assays and 0.2 µM in the *pfg27* and *pf40S* assays. The final reaction contained 1X TaqMan™ Universal PCR Master Mix No AmpErase™ UNG (Applied Biosystems,UK). The temperature profile was; 2 min at 50ºC, 10 min at 95ºC, and then 45 cycles of 15 s at 95ºC and 1 min at 60ºC. Quantification was carried out in duplicate with < 0.35 standard deviation between replicates.

#### qRT-PCR standard curves

To provide enough material for qRT-PCR optimization, establishment of sensitivity and for sample quantification assays, standard curves were based on cDNA (hereafter named ivcDNA) that was synthesised from RNA previously transcribed in vitro from DNA templates. DNA templates for each gene were prepared by PCR amplification of the target regions from *P. falciparum* clone 3D7 incorporating primers containing SP6 or T7 RNA polymerase promoter sequences (Table S2). Amplicons were purified from excess reagent and primers using Wizard SV Gel and PCR Clean-Up System (Promega, UK) as described by the manufacturer, and the concentration was determined using nanodrop (NanoDrop Spectrophotometer, ND1000, Thermo Fisher Scientific, UK). The number of molecules of DNA amplicon was calculated using the equation (ng DNA × 6.022 × 10^23^) / length (bp) of the DNA template × 10^9^ × 650. Purified DNA amplicon was then transcribed to RNA using Riboprobe Combination System-SP_6_/T_7_ RNA Polymerase (Promega), UK as described by the manufacturer. gDNA was removed from RNA using 1 unit of RQ1 DNase (Promega, UK) per 8 µL of RNA and qPCR was carried out to confirm the purity of the RNA prior to cDNA preparation. ivcDNA was prepared by RNA reverse transcription using High-Capacity cDNA Reverse Transcription Kit (Applied Biosystems, UK), USA as described by the manufacturer. The concentration of the ivcDNA was calculated based on the initial concentration of DNA amplicon and incorporating the dilution factors occurred during the processes above (DNA transcription, RNA purification and RNA reverse transcription). ivcDNA was then used to generate standard curves to assess qRT-PCR efficiency and sensitivity and to quantify the transcript number of the early gametocyte candidate genes in clinical samples.

#### Quantification of gametocyte gene expression and gametocyte numbers

The quantity of purified early gametocyte culture cDNA that was available was insufficient to run a quantitative standard curve against all clinical samples, thus an ivcDNA standard curve was used. The limit of quantification (LoQ) for the qRT-PCR assays was defined as the lowest ivcDNA concentration detectable in all experiments that fell within the log-linear relationship of the qRT-PCR assay^38^. Detection of ivcDNA concentrations below the LoQ was possible in > 50% of the standard curves. Although points below the LoQ were outside the log-linear relationship of the qRT-PCR, and could not be reliably quantified, they were considered to represent reliable detection because they fell within a clear amplification curve, in contrast to the cDNA-free sample (negative control)^39^. Thus, we considered these points as a reliable detection of early/late gametocytes but with lower certainty around their estimated gametocyte numbers as highlighted in the results and the discussion.

The transcript number of the stage-specific gametocyte genes was quantified in clinical samples based on the log-linear mathematical relationship between qRT-PCR cycle threshold (C_T_) and the log10 of the concentration of the ivcDNA standard curve (supplementary Figure S1). Then, the log-linear mathematical relationship between the transcript numbers and the early gametocyte numbers in the purified stage II gametocytes culture was established (supplementary Figure S2). The number of gametocytes/mL of blood in natural infections was estimated based on the average number of transcripts obtained from known numbers of purified stage II or stage V gametocytes, assuming 100% efficiency of the processes of reverse transcription, RNA purification and RNA extraction.

### Statistical analysis

Intra- and inter-assay coefficients of variation of the standard curves were calculated as the standard deviation of the C_T_ values divided by their mean C_T,_ and then multiplied by 100. The levels of the reference gene expression between different gametocyte stages were compared using Student’s t test. A Wilcoxon test was used to compare the relative expression of early and late gametocyte marker among stage II, stage V and asexual samples. Logistic regression was used to investigate the probability of detecting early gametocytes at variable levels of parasitaemia. Spearman’s correlation was used to assess the correlation between *pfpeg4* and *pfg27* expression in field samples. It was also used to assess the correlation between early and late gametocyte gene expression in field samples. R version 3.2.3 (2015-12-10) was used for statistical analysis.

## Results

### In silico analysis of gametocyte markers and the reference gene

To identify putative early-stage gametocyte markers, we searched published *P. falciparum* stage-specific gametocyte transcription analyses and transcriptome data^8,10,15,37,40–42^. Candidate genes were prioritised by comparing the transcript levels of fragments per kilobase of exon model per million mapped reads (FPKM) as described^43^. The genes *pfpeg4, pfg27, pfge1*, *pfge3* and *pfgexp5* were selected for evaluation in Taqman qRT-PCR assays. The gene *pfs16* was used as a pan-gametocyte stage marker, since Pfs16 protein expression starts 24–30 h post-invasion of a sexually-committed merozoite and continues throughout gametocyte development^40^. *P. falciparum* transcription data available in PlasmoDB were also searched to identify a candidate reference gene; *pfs40S* was selected as it is constitutively expressed, at a similar high level^44^, in ring-stage asexual parasites and stage II and V gametocytes, and could be used to estimate the relative expression of early gametocyte candidate genes.

### Efficiency, reproducibility, and sensitivity of qRT-PCR assay

The efficiency, sensitivity and reproducibility of the early-stage gametocyte qRT-PCR assays was assessed using ivcDNA of *pfpeg4*, *pfg27*, *pfge1*, *pfge3* and *pfs16,* and a late-stage gametocyte culture was used to test the *pfs25* qRT-PCR assay. In 4–6 independent experiments, the average qRT-PCR efficiency for the genes ranged between 85.6% and 92.2% and the maximum intra- and inter-assay coefficient of variation (CV) was 0.9%—6.4% and 1.9%—6.6% (Table S3 and Figure S1). In all cases, the low CV (< 10%) demonstrates the high reproducibility of the assays. The LoQ of the *pfpeg4, pfg27, pfge1, pfge3*, *pfs16* and *pfs25* qPCR assays was 0.14, 0.28, 0.2, 0.14, 0.68 and 0.15 gametocyte/µL of cDNA, which is equivalent to 0.56, 1.12, 0.8, 0.56, 2.72 and 0.60 gametocytes per qRT-PCR assay, respectively.

### Validation of early markers in purified stage II and V gametocytes

To establish that the expression level of the reference *pf40S* is similar across early and late gametocytes, qRT-PCR was performed*.* The reference gene, *pf40S,* showed similar expression levels (t-test T = 1.25, P = 0.28) in samples containing equal numbers of purified stage II (mean C_T_ = 38.73, SD = 0.67, in 4 technical replicates of 1 sample) and stage V gametocytes (mean C_T_ = 37.60, SD = 1.43, in 4 technical replicates of 1 sample).

**Figure 1:**
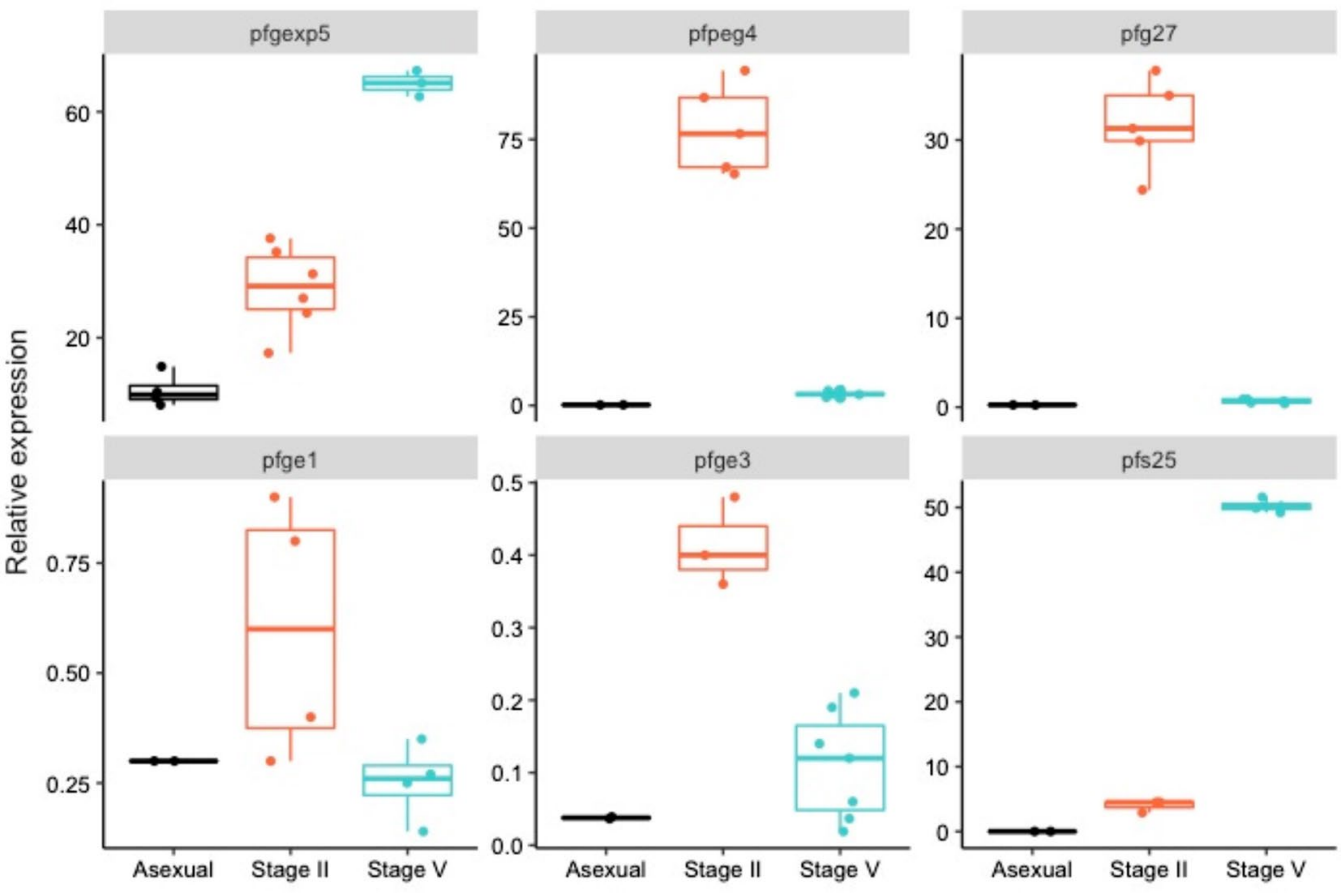
Validation of early gametocyte markers. Figure shows fold change (y axis) in expression of early (*pfpeg4*, *pfg27*, *pfge1*, *pfge3* and *pfgexp5*) and late (*pfs25*) markers relative to the reference gene (*pf40S*) expression in parasites obtained from in vitro culture at different parasite developmental stages. Points represents actual data points, boxplots represent median, first and third quartiles. Error bars represent the minimum and the maximum fold change.

The stage specificity of the early gametocyte genes was assessed by quantifying their expression in comparison to the reference gene *pf40S* in three samples of *P. falciparum* culture: (i) mixed asexual stages (ii) purified stage II gametocytes and (iii) purified stage V gametocytes. The relative expression of *pfpeg4, pfg27, pfge1*, *pfge3* and *pfgexp5* was examined in purified stage II, stage V gametocytes (4–6 technical replicates each), and in mixed asexual stages (3–4 technical replicates). Significantly higher expression of *pfpeg4* (~ 24-fold) and *pfg27* (~ 43-fold) was observed in stage II compared to stage V gametocytes (Wilcoxon signed-rank test, *pfpeg4*; P = 7.9 × 10^−3^; *pfg27*, P = 0.01), with negligible expression in asexual stages (Fig. 1). *Pfge1* and *pfge3* were expressed at higher levels in stage II compared to stage V*,* but their relative expression in stage II was considerably lower than *pfpeg4* (125-fold and 187-fold lower, respectively) and *pfg27* (50-fold and 75-fold lower, respectively). In contrast, *pfgexp5* was highly expressed in stage II gametocytes compared to asexual stages, but at higher levels in stage V gametocytes (Wilcoxon signed-rank test, P = 0.02). As expected, a significantly higher relative expression of *pfs25* (~ 11-fold) was observed in stage V compared to stage II gametocytes (Wilcoxon signed-rank test, P = 7.9 × 10^−3^, Fig. 1). Therefore, *pfpeg4* and *pfg27* were selected as early gametocyte markers for further validation in clinical samples.

### Detection of early and late stage gametocytes in natural *P. falciparum* infections.

**Figure 2:**
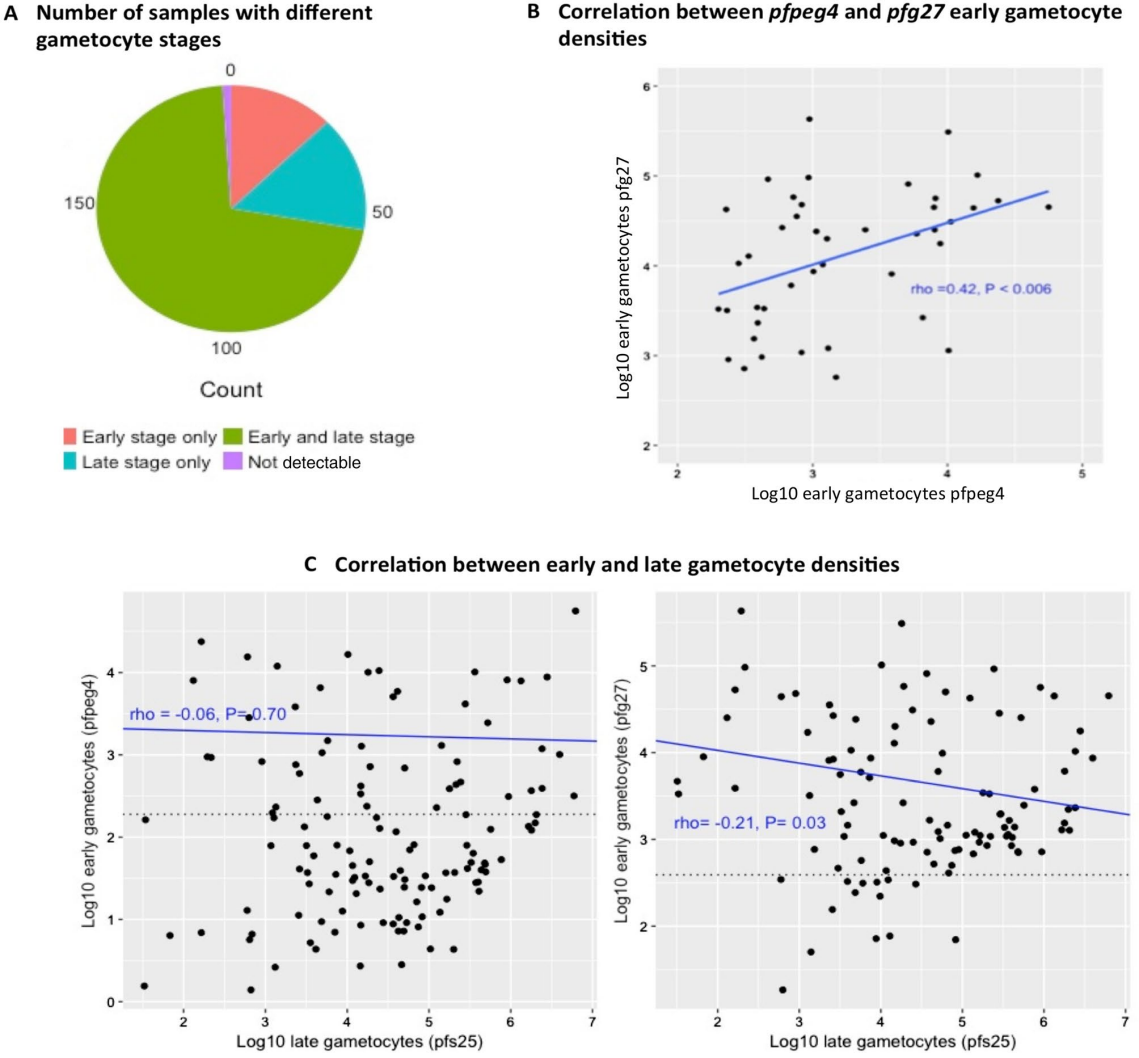
Early gametocytes in natural infections. (**A**) Number of samples with different gametocyte stages in field samples (n = 198). Early gametocyte detection is based on both *pfpeg4* and *pfg27* expression. (**B**) Correlation between log10 early gametocyte densities as detected by *pfpeg4* and *pfg27*. The figure shows correlations in samples where densities were above the LoQ. A figure demonstrating the correlation in all samples is given as supplementary Figure S4. (**C**) Correlation between log10 early (*pfpeg4* or *pfg27*) and late stage gametocytes/ ml of blood among samples with mixed stages. The figure shows that early stage gametocytes densities are independent, particularly for *pfpeg4*, to the density of late stage gametocytes. The dotted line in both panels represents the LoQ. Correlation line, Spearman correlation coefficient (rho) and P value were calculated for points above the LoQ. Points below LoQ are robustly detectable however with higher uncertainty around the estimated quantities.

Two hundred and fifty samples from patients with natural *P. falciparum* infections^31^ were used to investigate the specificity of the early gametocyte assays, across a range of parasitaemias, and in the presence of late gametocytes. Of the 250 samples, 79.2% (n = 198) were positive for *pfs16*, indicating the presence of gametocytes (supplementary Table S4). Of those 198 samples, 25 (12.6%) were positive for *pfpeg4* and/or *pfg27* but not for *pfs25,* indicating the presence of early gametocytes only, and 30 (15.2%) were positive for *pfs25* and not *pfpeg4* and *pfg27*, indicating the presence of only late gametocytes (Fig. 2A). The majority of the samples contained mixed early and late gametocyte stages (n = 141, 71.2%). However, for 2 (1%) samples out of the 198 that were *pfs16-*positive no transcripts for either early or late gametocyte genes were detected.

The *pfpeg4* and *pfg27* qRT-PCR assays detected early gametocytes in patients with a wide range of total parasitaemia, 79 to 2.6 × 10^8^ parasites/mL of blood as determined by 18 s rRNA qPCR. The presence of early stage gametocytes was associated with parasitaemia: for each tenfold increase in parasite density the probability of detecting early gametocytes increased by a factor of 2.22 (95% CI = 1.63–3.01) for *pfpeg4* and 2.12 (95% CI = 1.67–2.70) for *pfg27*. Of note, > 80% of samples containing early gametocytes were from patients with parasite density of > 10^4^ parasites/mL of blood (Figure S3).

The *pfpeg4* and *pfg27* qRT-PCR assays detected variable densities of early gametocytes, with a range of 1 to 5.6 × 10^4^ and 18.4 to 4.3 × 10^5^ early gametocytes/mL of blood, respectively. However, the densities of 104 and 19 samples were below LoQ for *pfpeg4* and *pfg27*, respectively (in Material and Methods section "Quantification of gametocyte gene expression and gametocyte numbers"). The LoQ of the assays was estimated as 0.14 and 0.28 early gametocyte/µL of cDNA for *pfpeg4* and *pfg27*, equivalent to 190 and 390 early gametocytes/mL of blood, respectively, assuming 100% efficiency of the RNA extraction, purification, and cDNA processes. A moderate correlation was seen between the early gametocyte densities quantified by *pfpeg4* and *pfg27* qRT-PCR assays (Spearman’s correlation coefficient (rho) = 0.63, P < 0.01, Figure S4). However, where early gametocyte densities were above the LoQ, this correlation was lower (rho = 0.40, P < 0.008, Fig. 2B).

Among samples containing a mix of early and late gametocytes and where early gametocyte densities were above the LoQ, early gametocyte densities did not correlate (*pfpeg4*: rho = 0.06, P = 0.70) or weakly correlated (*pfg27*: rho = − 0.21, P = 0.03) with densities of late stage gametocytes and detected by *pfs25* (Fig. 2C).

### Detection of early stage gametocytes in experimentally infected volunteers

**Figure 3:**
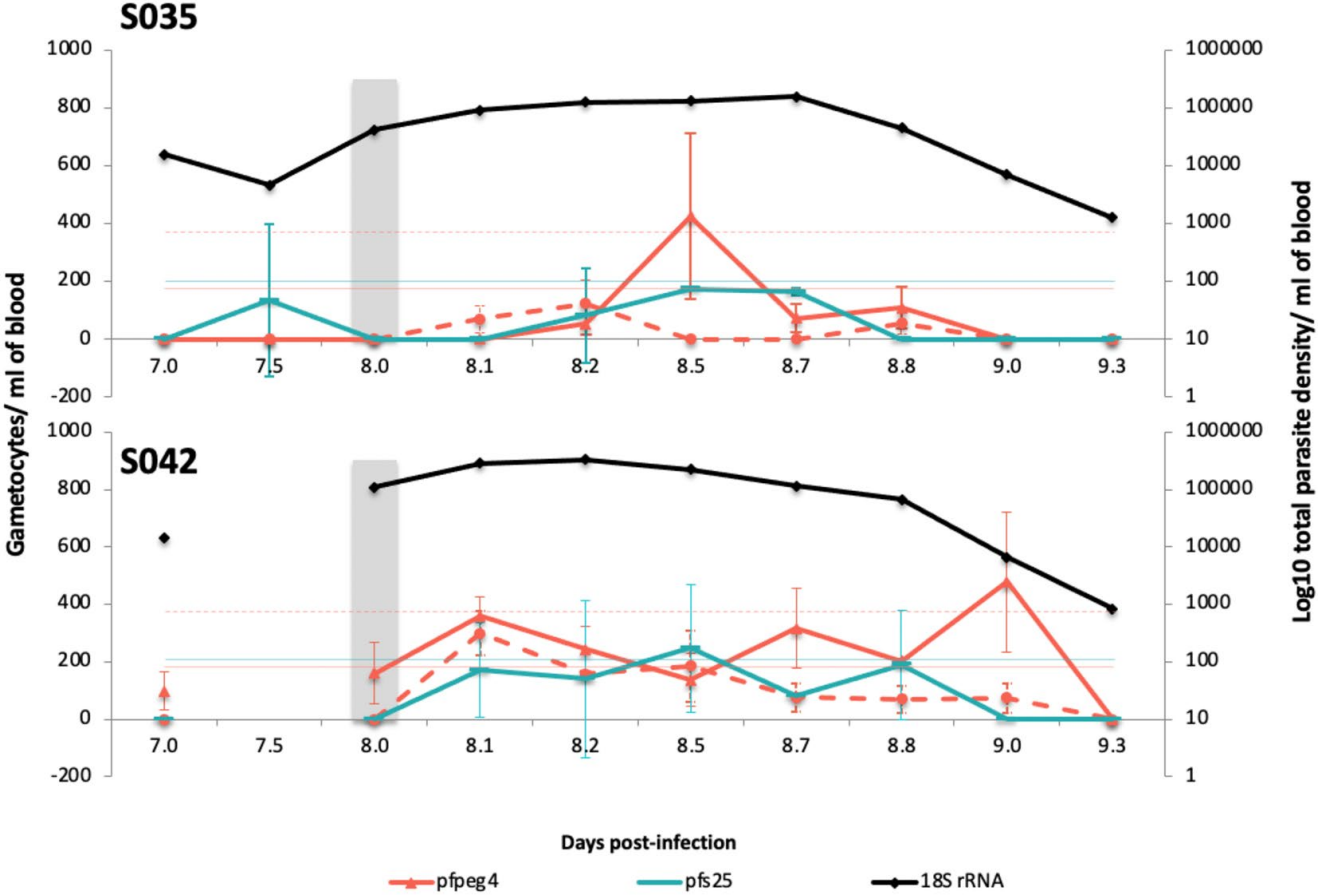
Detection of early gametocytes in vivo in human experimental infections. Left Y axis represents number of gametocytes/mL of blood, quantified by *pfpeg4*, *pfg27* or *pfs25* qRT-PCR. On the right Y axis is plotted total parasite density as quantified by *18S rRNA* qPCR. Horizontal lines represent limit of quantification of early and late gametocytes and reads to the left Y axis. Data shown is from 2 volunteers (S035 and S042) during follow up time-points between D07 and D09 post-infection. The X axis represents day post-infection and digits after the decimal points are fractions of that day. Volunteers were treated with piperaquine at D08 (grey shaded column). Data is provided in supplementary file (Table S5).

The specificity of the *pfg27* and *pfpeg4* assays was further tested in two volunteers experimentally infected with *P. falciparum*^15,32^. The parasite density ranged between 1.3 × 10^3^ and 1.6 × 10^5^ (median 4.4 × 10^4^) parasites/mL blood in volunteer S035 and 3.3 × 10^5^–8.4 × 10^2^ (median 5.4 × 10^4^) parasites/mL blood in volunteer S042 (Fig. 3) over days 7 to 10 post-infection. *pfpeg4* and *pfg27* transcripts were detectable at an early stage of the infection, day 8 and day 9 post infection (Fig. 3). Early gametocyte density (as identified by the *pfpeg4* and *pfg27* assays) fluctuated at low levels, often below the LoQ and with overlapping 95% CI throughout the follow up time points (*pfpeg4* range: 106–850; *pfg27* range: 113–371 early gametocytes /mL of blood). Slight differences in the detection profile of *pfpeg4* and *pfg27 *may reflect differences in the stage at which they are transcribed. In contrast, late gametocytes were only detected below the LoQ using the *pfs25* assay in the two volunteers and the detection of late gametocytes was associated with wide 95% CI, often crossing the zero due to detection occurring in only one of the sample duplicates (Fig. 3).

## Discussion

The present study describes sensitive qRT-PCR assays for the detection and quantification of early-stage gametocytes of *P. falciparum*. We initially examined the relative expression of five early gametocyte markers (*pfpeg4, pfg27, pfge1*, *pfge3* and *pfgexp5*) in purified early (stage II) and late (stage V) gametocyte and demonstrated that *pfpeg4* and *pfg27* are expressed at high levels only in the early stages, while *pfge1* and *pfge3* are expressed at low levels in stage II. In contrast, *pfgexp5* is expressed at high levels in both stage II and stage V gametocytes, and therefore is unable to distinguish between early- and late-stage gametocytes. Transcript quantification of gametocyte genes was estimated relative to that of the reference gene *pf40S*, which is expressed at the same level in stage II and V gametocytes of *P. falciparum*. Thus, variations in the amount of the starting transcripts were controlled for. The sensitivity of the *pfpeg4* and *pfg27* qRT-PCR assays were equivalent to detection thresholds of 190 and 390 gametocytes per mL of blood, respectively.

The specificity of the *pfpeg4 and pfg27* qRT-PCR assays was tested in 198 natural *P. falciparum* infections ,with gametocytes detectable by *pfs16*, from eastern Sudan^31^. The *pfpeg4* and/or *pfg27* transcripts were detected in 25 (12.6%) isolates in absence of the *pfs25* transcripts*,* suggesting the presence of a cohort of circulating early gametocytes when mature gametocytes were not present. Conversely, *pfs25* transcripts were detected in 30 (15.2%) isolates in the absence of *pfpeg4* and *pfg27* transcripts, indicating the presence of a circulating population comprising only late gametocytes (Fig. 2A). Nonetheless, the majority of the infections contain a mixture of early and late gametocyte stages (Fig. 2A). Evidence of circulating early stage *P. falciparum* gametocytes in natural infections is scarce^6,45^, but has been documented in cases of exceedingly high parasite burdens, when asexual stages that usually sequester are also visible in circulation, as well as in splenectomized infected individuals^46^.

Recent transcriptome analyses have identified a number of early gametocyte-specific transcripts expressed in sexually-committed ring stages^47^, that were also found in peripheral blood samples in natural infections^48^. The authors reported a high proportion of infections with circulating sexually-committed ring stage parasites (76%), but the ratio of gametocytes to sexually committed rings (in vitro) varied dramatically, ranging from 78% to absent. Prajapati et al.^49^ also reported a high prevalence (57.14%) of early gametocytes in children with asymptomatic *P. falciparum* infection in Ghana. Although gametocytes were not quantified in these reports, the pattern of detection of early gametocytes is consistent with that seen in our field setting in eastern Sudan, where 83.8% of infected individuals contained transcripts of early gametocyte stages (*pfpeg4* and/or *pfg27*).

These data suggest that, in addition to sexually committed ring stages, slightly older stage I gametocytes may also be found in the peripheral circulation. Further longitudinal analysis of asymptomatic infections, in the absence of re-infection in a highly seasonal endemic setting, such as that in eastern Sudan^50^ will elucidate epidemiological factors that drive gametocytogenesis. A previous cohort survey of asymptomatic infection in this region demonstrated an increase in *pfs25* transcripts, indicative of mature gametocytes, following resurgence of mosquitoes at the start of the rainy season. However, no corresponding increase in the density or prevalence of total parasites or gametocytes was seen^34^. Analysis of early gametocyte markers will enable verification of the hypothesis that *P. falciparum* may respond to environmental cues, such as mosquito biting, to modulate its transmission strategy^51–53^.

The robustness of the *pfpeg4* and *pfg27* assays is demonstrated by the ability to detect transcripts at a wide range of parasitaemia in field samples, from very high (2.6 × 10^8^ parasites/mL blood), always associated with clinical presentation, to sub-microscopic levels (79 parasites/mL blood), often present in asymptomatic infections^34,54,55^. The detectability of *pfpeg4* and/or *pfg27* transcripts is strongly linked to the level of total parasitaemia, in line with the performance of other qRT-PCR assays for gametocyte quantification; *pfgexp5*^15^, *pfs230p*^56^ and *pfs25*^57^.

In addition, the sensitivity of the *pfpeg4* and *pfg27* RT-qPCR (190 and 390 gametocytes per mL of blood, respectively) is comparable to that reported in other assays for gametocyte specific markers, such as the female (*pfs25*) and male (*pfs230p*) gametocyte specific RT-qPCR assays, with a detection limits of 0.3 female and 1.8 male gametocytes/µL blood, respectively^56^. Similar RT-qPCR assays for putative early gametocyte markers *(ap2-g, surfin 13.1, and surfin 1.2),* have been described with a similar low limit of detection (2 gametocytes/µL blood), a level below the level theoretically required to infect a mosquito^49^.

The specificity of the *pfpeg4* and *pfg27* qRT-PCR assays was further tested by analysis of samples from volunteers experimentally infected with *P. falciparum*, taken at D07–D09 post-infection when only early but not late gametocyte stages are present. The volume of the initial inoculum was 1800 parasites^32^; equivalent to approximately 0.72 × 10^−10^ parasite/ red blood cell, precluding the detection of gametocytes at early time points following inoculation. However, transcripts of *pfpeg4* and *pfg27* were detected at several time points between D7 and D9 (Fig. 3), often above the LoQ, demonstrating the detectability of circulating early gametocytes even at a low density and at this early stage of infection. In contrast, *pfs25* transcripts were detected mostly below the LoQ and with a wide 95% CI of replica including zero (Fig. 3).

In summary, the *pfpeg4* and *pfg27* qRT-PCR assays described here are specific and sensitive, and can quantify early *P. falciparum* gametocytes, as low as 190 and 390 gametocytes per mL of whole blood, respectively. The late-stage specificity assay (*pfs25*), currently used for field surveys, quantifies late gametocytes^16^. These early stage-specific qRT-PCR assays will enable studies aimed at improving understanding of factors that modulate transmission, such as the impact of drugs with various mechanisms of action, the multiplicity of infection, as well as climatic variables that influence transmission into the mosquito vector. In addition, it will enable novel epidemiological and biological investigations, such as evaluation of ongoing gametocyte production after exposure to various antimalarials, the proportion of early gametocytes in an individual that progress to mature transmission competent gametocytes, identification of spatial variability in transmission, and the effect of a range of interventions intended to reduce transmission. A better understanding of the gametocyte reservoir in natural infections is essential for design of novel approaches for malaria elimination, and for the assessment of novel transmission-blocking tools.

## Supplementary Information

Supplementary Information.

## Data Availability

All data generated or analysed during this study are included in this published article (and its Supplementary Information files).
