# Supplementary material for: Real-time PCR assays for detection and quantification of early P. falciparum gametocyte stages
Source: Sci Rep. 2021 Sep 27;11:19118. doi: 10.1038/s41598-021-97456-4 (PMC8476600; doi:10.1038/s41598-021-97456-4)
Supplement: Supplementary file 1 — Supplementary Information. [file 41598_2021_97456_MOESM1_ESM.pdf]

Table S1: Genes names and Accession Numbers

Gene name	Annotation	Old ID	Current Accession #
18s rRNA	18S ribosomal RNA	-<br>MAL11_rRNA<br>-	PF3D7_0112300<br>PF3D7_1148600<br>PF3D7_1371000
pfs16	Sexual stage-specific protein precursor	MAL4P1.61, PFD0310w	PF3D7_0406200
pfgexp5	Gametocyte exported protein 5	-	PF3D7_0936600
pfpeg4	Early transcribed membrane protein 10.3 (ETRAMP10.3)	PF10_0164	PF3D7_1016900
pfg27	Gametocyte specific protein 27/25	PF13_0011	PF3D7_1302100
pfge1	Gametocytogenesis early gene 1	PF14_0744	PF3D7_1477300
pfge2	Gametocytogenesis early gene 2	PF14_0745	PF3D7_1477400
pfge3	Gametocytogenesis early gene 3	PF14_0748	PF3D7_1477700
pfs25	25 kDa ookinete surface antigen precursor	PF10_0303	PF3D7_1031000
Pf40S	40S ribosomal protein S3	PF14_0627	PF3D7_1465900

Table S2: Primers and probes sequencing. Primers and TaqMan dual labeled probes used for quantification of total parasite density, early gametocytes and late gametocytes. Fw = Forward and Rv = reverse, dual labeling in probes sequence indicated in bold, FAM = 6-carboxyfluorescein. Oligonucleotide shaded in blue were used to insert T7 or PS6 promoter regions for the construction of the ivcDNA materials.

Oligo name	Oligonucleotide sequence (5` to 3`) and label
pfs16 Qt.Fw	CCTTCAACTTTGCAAACCC
pfs16 Qt.Rv	GCTAGCTGAGTTTCTAAAGGCAT
pfs16 Qt.Pr	**Fam**-TGCCTCTCTTCATGCTGTTGGACC-**Tamra**
pfg10.164 QtFw	GCGTTGAAGGATATCGACAG
pfg10.164 QtRv	CCTAAACTTCCAAATAAACCACCT
pfg10.164 QtPr	**Fam**-TGCTGCTGTTGCTTTGGCTATAACCTT- **Tamra**
SP6_0164Fw1	GACACTATAGGTTTCTAGGCATACCGTTC
SP6_0164Fw2	GATTTAGGTGACACTATAGGTTTCTAGGCATACCGTTC
0164Rv	AGAGTCGGATCCATCATTCTC
pfg27 QtFw	GCGTATCATGAACGACAAGAA
pfg27 QtRv	ACGGGTAAAGCAGGTATTGG
pfg27 QtPr	JOE-TGACCAATATAAGGACGCAGCAGCA-Tamra
T7-Pfg27Fw1	ACTATAGGGTTAGTATCAGGAGATATGTTCG
T7-Pfg27Fw2	TAATACGACTCACTATAGGGTTAGTATCAGGAGATATGTTCG
Pfg27Rv	GTTGTGATGTGGTTCATCAGGTG
GEXPFWD	CTTCTTGTTCGAGATTATCCCT
GEXPREV	GGAGTCTACTAATTCAGACAGC
GEXPPROBE	**JOE** -TGTAATGTAGTAGAAGGTACCATTGGTCA-**Tamra**
pfg14.744 QtFw (*Pfge1*)	TAATGGCTCTGTTGACGGAA
pfg14.744 QtRv (*Pfge1*)	TGAAGAAGAATAATTTGCAGAAGC
pfg14.744 QtPr (*Pfge1*)	**JOE** -TCAGAGAGGCTTCTCGACTTCCTCG-**Tamra**
pfg14.748 QtFw (*Pfge2*)	TCACATAATGAATTCAAGGGTAGTT
pfg14.748 QtRv (*Pfge2*)	TCTTCAGCTTTATCAGAATTCCC
pfg14.748 QtPr (*Pfge2*)	**Fam**-CTCTGATTTGGCCACACTGCTCTAGG-**Tamra**
pfs25 Qt.Fw	GACTGTAAATAAACCATGTGGAGATT
pfs25 Qt.Rv	CCGTTACCACAAGTTACATTCTTAC
pfs25 Qt.Pr	**FAM**- TGGAAATCCCGTTTCATACGCTTGT-T**amra**
Pf40s.Qt.Fw	TGCTGAAAGAGTAGAACATAGAGGA
Pf40s.Qt.Rv	TCCTTTAGCACCAGATTCCA
Pf40s.Qt.Pr	**FAM**- TCGGCTTGTGCCATTGCACA-T**amra**

Table S3. Average qRT-PCR efficiency and maximum intra- and inter-assay coefficient of variation (4-6 experiments) for the genes *pfpeg4*, *pfg27*, *pfge1*, *pfge3*, *pfs16*, *pfs25*.

	Average qRT-PCR efficiency % (SD)	intra-assay coefficient of variation %	Inter-assay coefficient of variation %
pfpeg4	88.3 (13.3)	4.2	2.7
pfg27	85.6 (4.9)	2.4	4.0
pfge1	92.2 (12.8)	6.4	5.9
pfge3	81.3 (7.15)	2.3	6.6
pfs16	90.7 (11.9)	0.9	1.9
pfs25	88.2 (0.57)	1.2	3.8

Table S4: Raw data for field samples

PID	Total parasite	Q.Total parasite	Any Gametocyte	Early (pfpeg4)	Q.Early (pfpeg4)	Early (pfg27)	Q. Early (pfg27)	Late (pfs25)	Q.Late (pfs25)	Gametocytes profile
E01/0	Yes	22981726.6	Yes	Yes	10086.3	Yes	308052.8	Yes	18014.5	Early and late gametocytes
E01/07	Yes	74935.0	No	No	0.0	No	0.0	No	0.0	pfs16 negative
E01/14	Yes	5188529.3	Yes	Yes	16632.2	Yes	102216.6	Yes	10205.0	Early and late gametocytes
E01/21	Yes	561040.6	Yes	Yes	8.4	Yes	243.9	Yes	4855.9	Early and late gametocytes
E03/0	Yes	811955.4	Yes	Yes	1.8	Yes	2492.3	No	0.0	Early gametocytes only
E03/07	Yes	18696.3	Yes	No	0.0	No	0.0	Yes	254.7	Late gametocytes only
E03/14	Yes	10081.8	Yes	Yes	32.3	Yes	711.8	Yes	36978.5	Early and late gametocytes
E03/21	Yes	3304.0	Yes	No	0.0	No	0.0	Yes	1366.4	Late gametocytes only
E04/0	Yes	15061936.5	Yes	Yes	929.5	Yes	96052.4	Yes	215.8	Early and late gametocytes
E04/07	Yes	3758869.1	Yes	Yes	11.9	Yes	344.7	Yes	597.0	Early and late gametocytes
E04/14	Yes	1995046.0	Yes	Yes	7928.9	Yes	44894.2	Yes	1335742.1	Early and late gametocytes
E04/21	Yes	301099.7	Yes	Yes	11.6	Yes	71.7	Yes	8754.6	Early and late gametocytes
E07/0	Yes	2656414.1	Yes	Yes	5.9	Yes	3887.8	Yes	163.6	Early and late gametocytes
E07/07	Yes	6041387.7	Yes	Yes	44.1	Yes	436.4	Yes	11639.2	Early and late gametocytes
E07/14	Yes	1017725.2	Yes	Yes	690.3	Yes	6061.5	Yes	50415.5	Early and late gametocytes
E07/21	Yes	1616603.8	Yes	Yes	58.3	Yes	326.9	Yes	3897.1	Early and late gametocytes
E08/0	Yes	14059118.2	Yes	No	0.0	Yes	6498.9	No	0.0	Early gametocytes only
E08/07	Yes	341.1	No	No	0.0	No	0.0	No	0.0	pfs16 negative
E08/14	Yes	202225.1	Yes	Yes	435.2	Yes	3350.8	Yes	214465.0	Early and late gametocytes
E08/21	Yes	207369.2	Yes	No	0.0	No	0.0	Yes	94.5	Late gametocytes only
E9/0	Yes	5441178.7	Yes	Yes	115.4	Yes	1665.7	Yes	39599.3	Early and late gametocytes
E9/07	Yes	2401.1	Yes	No	0.0	No	0.0	Yes	10586.5	Late gametocytes only
E9/14	Yes	1057.3	No	No	0.0	No	0.0	Yes	13302.4	pfs16 negative
E9/21	Yes	2024.9	No	No	0.0	No	0.0	Yes	5295.4	pfs16 negative
E11/0	Yes	6218954.1	Yes	Yes	35.0	Yes	371.2	No	0.0	Early gametocytes only
E11/07	Yes	102429.6	Yes	Yes	9.7	Yes	69.8	Yes	82598.1	Early and late gametocytes
E11/14	Yes	233765.9	Yes	Yes	22.5	No	0.0	Yes	25225.9	Early and late gametocytes
E11/21	Yes	23548.4	Yes	No	0.0	No	0.0	Yes	5645.0	Late gametocytes only
E13/0	Yes	2794108.4	Yes	Yes	0.5	Yes	4670.6	Yes	32.0	Early and late gametocytes

E13/07	Yes	272204.6	Yes	Yes	1.6	No	0.0	Yes	1314.1	Early and late gametocytes
E13/14	Yes	227.2	No	No	0.0	No	0.0	No	0.0	pfs16 negative
E15/0	Yes	92257492.2	Yes	Yes	38.6	Yes	2709.0	No	0.0	Early gametocytes only
E15/07	Yes	382587.2	Yes	Yes	36.1	No	0.0	Yes	206261.8	Early and late gametocytes
E15/14	Yes	11425439.5	Yes	Yes	62.6	Yes	1088.0	Yes	346815.7	Early and late gametocytes
E15/21	Yes	6272318.4	Yes	Yes	38.2	Yes	519.8	Yes	44677.3	Early and late gametocytes
E16/0	Yes	71021820.3	Yes	Yes	1273.4	Yes	20074.7	Yes	14935.2	Early and late gametocytes
E17/0	Yes	19938337.9	Yes	Yes	36.0	Yes	2088.6	Yes	3269.7	Early and late gametocytes
E17/07	Yes	5833.8	Yes	No	0.0	No	0.0	Yes	50760.0	Late gametocytes only
E17/14	Yes	3986.6	Yes	No	0.0	No	0.0	Yes	23741.2	Late gametocytes only
E17/21	Yes	910.5	Yes	No	0.0	No	0.0	Yes	5135.0	Late gametocytes only
E18/0	Yes	25348947.3	Yes	Yes	8.0	Yes	1887.2	No	0.0	Early gametocytes only
E18/07	Yes	66497.6	Yes	Yes	7.7	No	0.0	No	0.0	Early gametocytes only
E18/14	Yes	13914888.7	Yes	Yes	23771.1	Yes	52983.9	Yes	163.6	Early and late gametocytes
E18/21	Yes	23341.5	Yes	No	0.0	No	0.0	Yes	418.6	Late gametocytes only
E19/0	Yes	413570.4	No	No	0.0	No	0.0	No	0.0	pfs16 negative
E19/07	Yes	9535.0	Yes	No	0.0	No	0.0	Yes	33213.4	Late gametocytes only
E19/21	Yes	61706.7	Yes	Yes	23.6	No	0.0	Yes	49980.6	Early and late gametocytes
E20/0	Yes	8283514.6	Yes	No	0.0	No	0.0	No	0.0	Fail to detect early or late markers
E20/07	Yes	7418296.4	Yes	Yes	8135.7	Yes	56485.3	Yes	902612.5	Early and late gametocytes
E20/14	Yes	14583251.0	Yes	Yes	8020.2	Yes	25200.9	Yes	130.4	Early and late gametocytes
E20/21	Yes	35074.3	Yes	Yes	79.8	Yes	1453.9	Yes	65675.8	Early and late gametocytes
E22/0	Yes	3407853.8	Yes	Yes	5.8	Yes	1982.2	No	0.0	Early gametocytes only
E22/07	Yes	2789.9	Yes	Yes	5.6	No	0.0	Yes	685.6	Early and late gametocytes
E22/14	Yes	1122.2	Yes	No	0.0	No	0.0	Yes	177.6	Late gametocytes only
E22/21	Yes	756.6	No	No	0.0	No	0.0	No	0.0	pfs16 negative
E23/0	Yes	25859468.8	Yes	Yes	77.9	Yes	5598.4	Yes	3175.0	Early and late gametocytes
E23/07	Yes	1315364.7	Yes	Yes	56.8	Yes	1188.1	No	0.0	Early gametocytes only
E23/14	Yes	2167722.2	Yes	Yes	26.0	No	0.0	Yes	3432.6	Early and late gametocytes
E25/0	Yes	23714128.9	Yes	Yes	170.7	Yes	17126.3	Yes	1268.8	Early and late gametocytes
E25/07	Yes	802809.6	Yes	Yes	310.3	Yes	718.1	Yes	938228.6	Early and late gametocytes
E25/14	Yes	676006.1	Yes	Yes	31.3	Yes	341.3	Yes	12311.8	Early and late gametocytes

E25/21	Yes	18198.8	Yes	Yes	26.9	No	0.0	Yes	18370.6	Early and late gametocytes
E26/0	Yes	18995660.2	Yes	Yes	40.0	Yes	8406.4	Yes	2611.6	Early and late gametocytes
E26/07	Yes	537.9	No	No	0.0	No	0.0	Yes	1093.0	pfs16 negative
E26/14	Yes	1223.0	Yes	No	0.0	Yes	321.4	Yes	8960.8	Early and late gametocytes
E26/21	Yes	728.8	No	No	0.0	Yes	592.2	Yes	7294.2	pfs16 negative
E27/0	Yes	9514878.9	Yes	Yes	10.6	Yes	16943.5	No	0.0	Early gametocytes only
E27/07	Yes	4003.0	Yes	No	0.0	No	0.0	Yes	8154.6	Late gametocytes only
E27/14	Yes	1441.8	Yes	No	0.0	Yes	305.2	Yes	26825.7	Early and late gametocytes
E27/21	Yes	612.5	Yes	No	0.0	No	0.0	Yes	11985.1	Late gametocytes only
E28/0	Yes	26853002.0	Yes	Yes	4.2	Yes	1082.8	Yes	3548.1	Early and late gametocytes
E28/07	Yes	1105.4	No	No	0.0	No	0.0	Yes	3780.7	pfs16 negative
E28/14	Yes	150.9	No	No	0.0	No	0.0	Yes	1867.4	pfs16 negative
E31/0	Yes	14779603.5	Yes	Yes	36.0	Yes	2052.3	No	0.0	Early gametocytes only
E31/07	Yes	1393.5	Yes	Yes	0.4	No	0.0	Yes	664.7	Early and late gametocytes
E31/14	Yes	2170.0	Yes	No	0.0	No	0.0	Yes	198.0	Late gametocytes only
E31/21	Yes	211.8	No	No	0.0	No	0.0	Yes	32.0	pfs16 negative
E32/0	Yes	27170927.7	Yes	No	0.0	No	0.0	No	0.0	Fail to detect early or late markers
E32/07	Yes	113172.6	Yes	Yes	6.0	No	0.0	Yes	7077.6	Early and late gametocytes
E32/14	Yes	5739809.6	Yes	Yes	126.6	Yes	928.6	Yes	24946.6	Early and late gametocytes
E32/21	Yes	257943.4	Yes	No	0.0	No	0.0	Yes	598.1	Late gametocytes only
E35/0	Yes	36096722.7	Yes	Yes	13.1	Yes	4461.3	No	0.0	Early gametocytes only
E35/07	Yes	1380.4	Yes	No	0.0	No	0.0	Yes	3780.7	Late gametocytes only
E35/14	Yes	1048.2	Yes	Yes	1.7	No	0.0	Yes	14455.9	Early and late gametocytes
E35/21	Yes	395.2	No	No	0.0	No	0.0	No	0.0	pfs16 negative
E36/0	Yes	15004209.0	Yes	Yes	15529.9	Yes	44185.2	Yes	602.3	Early and late gametocytes
E36/07	Yes	773661.3	Yes	No	0.0	Yes	316.7	No	0.0	Early gametocytes only
E36/14	Yes	5313979.0	Yes	Yes	236.3	Yes	905.4	Yes	17425.0	Early and late gametocytes
E36/21	Yes	695887.3	Yes	Yes	4.7	No	0.0	Yes	640.2	Early and late gametocytes
E37/0	Yes	11546363.3	Yes	Yes	176.7	Yes	5960.5	Yes	5694.5	Early and late gametocytes
E37/07	Yes	26738.5	No	No	0.0	No	0.0	No	0.0	pfs16 negative
E37/14	Yes	146800.3	No	No	0.0	No	0.0	Yes	4601.9	pfs16 negative
E37/21	Yes	11271.3	Yes	Yes	9.6	No	0.0	Yes	42812.4	Early and late gametocytes

E38/0	Yes	69345468.8	Yes	Yes	718.3	Yes	58075.2	Yes	19060.9	Early and late gametocytes
E38/07	Yes	177105.3	Yes	Yes	6.2	No	0.0	Yes	42293.2	Early and late gametocytes
E38/14	Yes	74797.1	Yes	Yes	123.5	Yes	2473.9	Yes	568738.6	Early and late gametocytes
E38/21	Yes	23878785.2	Yes	Yes	227.6	Yes	42464.7	Yes	123877.0	Early and late gametocytes
E39/0	Yes	94583562.5	Yes	Yes	6542.7	Yes	2642.3	Yes	4689.4	Early and late gametocytes
E39/07	Yes	14130.7	Yes	Yes	48.3	Yes	1375.0	Yes	328319.0	Early and late gametocytes
E39/14	Yes	11929.5	Yes	Yes	15.3	No	0.0	Yes	70863.2	Early and late gametocytes
E39/21	Yes	9494.6	Yes	Yes	11.2	Yes	679.7	Yes	136285.5	Early and late gametocytes
E43/0	Yes	20040695.3	Yes	Yes	78.8	Yes	8660.0	Yes	7520.7	Early and late gametocytes
E43/07	Yes	20197.8	Yes	Yes	8.1	No	0.0	Yes	27570.7	Early and late gametocytes
E43/14	Yes	2320.1	Yes	Yes	16.6	Yes	1108.8	Yes	165085.3	Early and late gametocytes
E43/21	Yes	393.3	No	No	0.0	No	0.0	No	0.0	pfs16 negative
E44/0	Yes	206369687.5	Yes	Yes	5900.0	Yes	22811.9	Yes	41237.3	Early and late gametocytes
E44/07	Yes	509719.6	Yes	Yes	23.3	No	0.0	Yes	107545.6	Early and late gametocytes
E44/14	Yes	37884140.6	Yes	Yes	2452.6	Yes	25273.2	Yes	522875.5	Early and late gametocytes
E44/21	Yes	53352.7	Yes	Yes	1184.4	Yes	10338.7	Yes	2415962.2	Early and late gametocytes
E45/0	Yes	4398569.3	Yes	No	0.0	No	0.0	Yes	2825.4	Late gametocytes only
E45/07	Yes	6260.0	Yes	No	0.0	No	0.0	Yes	50564.0	Late gametocytes only
E45/14	Yes	3535.6	Yes	Yes	8.1	Yes	1021.9	Yes	53351.0	Early and late gametocytes
E45/21	Yes	2838.4	No	No	0.0	No	0.0	Yes	12479.3	pfs16 negative
E46/0	Yes	8916243.2	No	No	0.0	No	0.0	No	0.0	pfs16 negative
E46/07	Yes	148162.5	Yes	Yes	386.6	Yes	3447.7	Yes	178556.2	Early and late gametocytes
E46/14	Yes	66629.2	Yes	Yes	7.5	No	0.0	Yes	14659.9	Early and late gametocytes
E46/21	Yes	23672.6	No	No	0.0	No	0.0	Yes	197.2	pfs16 negative
E51/0	Yes	20595726.6	Yes	Yes	759.2	Yes	35477.9	Yes	2348.8	Early and late gametocytes
E51/07	Yes	58892.8	Yes	Yes	3.3	Yes	850.2	Yes	200344.2	Early and late gametocytes
E51/14	Yes	4202.0	Yes	No	0.0	No	0.0	Yes	39014.1	Late gametocytes only
E51/21	Yes	1931.2	No	No	0.0	No	0.0	No	0.0	pfs16 negative
E52/0	Yes	134177.7	Yes	Yes	120.5	Yes	6104.4	Yes	1792258.9	Early and late gametocytes
E52/07	Yes	9374.4	Yes	Yes	21.0	Yes	1056.7	Yes	408504.9	Early and late gametocytes
E52/14	Yes	1313.9	Yes	Yes	35.9	Yes	929.3	Yes	160777.0	Early and late gametocytes
E52/21	Yes	1671.4	Yes	No	0.0	No	0.0	Yes	41024.0	Late gametocytes only

E54/0	Yes	3480140.6	Yes	Yes	23.6	Yes	10980.2	No	0.0	Early gametocytes only
E54/07	Yes	12094.4	No	No	0.0	No	0.0	No	0.0	pfs16 negative
E54/14	Yes	14068.4	Yes	Yes	47.5	Yes	721.1	Yes	479610.4	Early and late gametocytes
E54/21	Yes	913.2	Yes	No	0.0	No	0.0	Yes	15036.1	Late gametocytes only
E55/0	Yes	14256019.5	No	No	0.0	No	0.0	No	0.0	pfs16 negative
E55/07	Yes	397.3	No	No	0.0	No	0.0	No	0.0	pfs16 negative
E55/14	Yes	753.0	No	No	0.0	No	0.0	No	0.0	pfs16 negative
E56/0	Yes	16307435.5	Yes	Yes	5.4	Yes	8946.1	Yes	67.0	Early and late gametocytes
E56/07	Yes	581595.8	Yes	Yes	56101.2	Yes	45166.3	Yes	6202978.6	Early and late gametocytes
E56/14	Yes	135019.7	Yes	Yes	8833.7	Yes	17747.0	Yes	2786060.3	Early and late gametocytes
E56/21	Yes	20160.6	Yes	Yes	32.6	No	0.0	Yes	16666.4	Early and late gametocytes
E57/0	Yes	69551710.9	Yes	Yes	592.5	Yes	26682.7	Yes	2609.7	Early and late gametocytes
E57/07	Yes	328.5	Yes	Yes	17.3	Yes	4976.0	No	0.0	Early gametocytes only
E57/14	Yes	3608.0	No	No	0.0	Yes	809.9	No	0.0	pfs16 negative
E57/21	Yes	908.9	Yes	No	0.0	Yes	766.3	Yes	1535.4	Early and late gametocytes
E59/0	Yes	6954930.7	Yes	Yes	186.5	Yes	28401.1	Yes	283199.5	Early and late gametocytes
E59/07	Yes	19932.9	No	No	0.0	No	0.0	Yes	1794.5	pfs16 negative
E59/14	Yes	1559.1	Yes	Yes	29.5	Yes	1228.1	Yes	50540.8	Early and late gametocytes
E59/21	Yes	2698.0	Yes	No	0.0	Yes	221.3	Yes	9796.4	Early and late gametocytes
E60/0	Yes	54627746.1	Yes	Yes	827.7	Yes	47934.1	Yes	903.9	Early and late gametocytes
E60/07	Yes	18678.6	Yes	Yes	20.6	Yes	312.7	Yes	6013.7	Early and late gametocytes
E60/14	Yes	1132091.2	Yes	Yes	1.1	No	0.0	No	0.0	Early gametocytes only
E60/21	Yes	1690.9	Yes	Yes	10.2	Yes	155.5	Yes	2550.1	Early and late gametocytes
E61/0	Yes	13537384.8	Yes	Yes	465.7	Yes	92143.9	Yes	243992.7	Early and late gametocytes
E61/07	Yes	21809.3	Yes	Yes	32.8	Yes	763.0	Yes	90314.8	Early and late gametocytes
E61/14	Yes	19594.6	Yes	Yes	78.5	Yes	1953.3	Yes	290359.8	Early and late gametocytes
E61/21	Yes	178769.2	Yes	No	0.0	No	0.0	Yes	3698.0	Late gametocytes only
E63/0	Yes	93298984.4	Yes	Yes	5084.2	Yes	81414.3	Yes	36498.0	Early and late gametocytes
E63/07	Yes	45834.2	Yes	No	0.0	Yes	521.7	No	0.0	Early gametocytes only
E63/14	Yes	10597.2	Yes	Yes	1009.1	Yes	8649.5	Yes	3958566.7	Early and late gametocytes
E63/21	Yes	413.4	Yes	Yes	23.5	Yes	741.7	Yes	80969.9	Early and late gametocytes
E66/0	Yes	38645589.8	Yes	Yes	2835.5	Yes	18.4	Yes	629.4	Early and late gametocytes

E66/07	Yes	1511541.4	Yes	Yes	4156.7	No	0.0	Yes	278132.5	Early and late gametocytes
E66/14	Yes	3536696.3	Yes	Yes	825.3	Yes	1084.0	Yes	220708.1	Early and late gametocytes
E66/21	Yes	11147.0	Yes	Yes	171.5	No	0.0	Yes	22866.7	Early and late gametocytes
E68/0	Yes	197907.5	Yes	Yes	38.9	Yes	1386.5	Yes	436387.3	Early and late gametocytes
E68/07	Yes	148815.5	No	No	0.0	No	0.0	No	0.0	pfs16 negative
E68/14	Yes	29705.4	Yes	Yes	40.6	Yes	1967.7	Yes	295348.9	Early and late gametocytes
E68/21	Yes	5079.6	Yes	No	0.0	Yes	409.9	Yes	67946.4	Early and late gametocytes
E69/0	Yes	2789264.2	Yes	Yes	13.6	Yes	958.1	No	0.0	Early gametocytes only
E69/07	Yes	62.8	No	No	0.0	No	0.0	No	0.0	pfs16 negative
E69/14	Yes	2167.0	No	No	0.0	No	0.0	No	0.0	pfs16 negative
E70/0	Yes	2457231.9	Yes	Yes	1060.9	Yes	24201.1	Yes	4919.1	Early and late gametocytes
E70/07	Yes	2215055.7	Yes	No	0.0	No	0.0	Yes	75633.7	Late gametocytes only
E70/14	Yes	27577.8	Yes	Yes	366.3	Yes	1541.8	Yes	1783436.9	Early and late gametocytes
E70/21	Yes	56413.2	Yes	Yes	390.4	Yes	2315.4	Yes	2418283.4	Early and late gametocytes
E71/0	Yes	12023570.3	Yes	Yes	231.0	Yes	3197.8	Yes	1340.0	Early and late gametocytes
E71/14	Yes	157.1	No	No	0.0	No	0.0	Yes	2415.5	pfs16 negative
E71/21	Yes	339.7	No	No	0.0	No	0.0	Yes	3470.2	pfs16 negative
E72/0	Yes	12060939.5	Yes	Yes	11961.0	Yes	50.4	Yes	1394.2	Early and late gametocytes
E72/07	Yes	2287.5	Yes	Yes	77.8	No	0.0	Yes	1166.3	Early and late gametocytes
E72/14	Yes	295.3	No	No	0.0	No	0.0	Yes	105.8	pfs16 negative
E72/21	Yes	455.3	No	Yes	17.7	No	0.0	Yes	18.9	pfs16 negative
E73/0	Yes	927685.4	Yes	Yes	132.3	Yes	465.4	Yes	3000.4	Early and late gametocytes
E73/07	Yes	1318.0	Yes	No	0.0	No	0.0	Yes	33899.6	Late gametocytes only
E73/21	Yes	231.7	No	No	0.0	No	0.0	No	0.0	pfs16 negative
E75/0	Yes	19933832.0	Yes	Yes	147.9	Yes	2213.9	Yes	1990302.2	Early and late gametocytes
E75/07	Yes	189805.1	Yes	Yes	186.8	Yes	1273.6	Yes	2056259.0	Early and late gametocytes
E75/14	Yes	109240.4	Yes	Yes	134.2	Yes	1284.4	Yes	1651845.3	Early and late gametocytes
E75/21	Yes	44827.7	Yes	Yes	45.5	Yes	703.2	Yes	485451.5	Early and late gametocytes
E78/0	Yes	2572113.0	Yes	Yes	281.6	Yes	10647.8	Yes	4311.9	Early and late gametocytes
E78/07	Yes	832.4	Yes	Yes	6.2	No	0.0	Yes	49096.3	Early and late gametocytes
E78/14	Yes	499.8	Yes	No	0.0	No	0.0	Yes	23957.2	Late gametocytes only
E78/21	Yes	863917.1	Yes	Yes	67.1	Yes	1108.9	Yes	10726.6	Early and late gametocytes

E79/0	Yes	1479102.4	Yes	Yes	7.1	Yes	502.0	Yes	74124.6	Early and late gametocytes
E79/07	Yes	768.9	Yes	Yes	1.8	No	0.0	Yes	46264.8	Early and late gametocytes
E79/21	Yes	54.2	No	No	0.0	No	0.0	No	0.0	pfs16 negative
E80/0	Yes	1417797.4	Yes	Yes	19.6	Yes	77.0	Yes	12920.4	Early and late gametocytes
E80/07	Yes	1070.7	No	No	0.0	No	0.0	No	0.0	pfs16 negative
E80/14	Yes	199.1	No	No	0.0	No	0.0	Yes	1803.1	pfs16 negative
E80/21	Yes	270.5	No	No	0.0	No	0.0	Yes	2038.8	pfs16 negative
E81/0	Yes	49606375.0	Yes	Yes	1485.8	Yes	571.9	Yes	5765.1	Early and late gametocytes
E81/07	Yes	5600.6	No	No	0.0	No	0.0	No	0.0	pfs16 negative
E81/14	Yes	348.3	No	No	0.0	No	0.0	Yes	6068.1	pfs16 negative
E82/0	Yes	2354426.3	Yes	Yes	52.3	Yes	3784.0	Yes	766903.0	Early and late gametocytes
E82/07	Yes	3258.0	Yes	Yes	3.4	No	0.0	Yes	104191.9	Early and late gametocytes
E82/14	Yes	46244.0	No	No	0.0	No	0.0	No	0.0	pfs16 negative
E82/21	Yes	407666.0	Yes	Yes	34.2	Yes	5143.0	Yes	7267.5	Early and late gametocytes
E84/0	Yes	296350.0	Yes	Yes	49.8	Yes	119637.3	No	0.0	Early gametocytes only
E84/14	Yes	18671.6	Yes	Yes	334.7	Yes	12831.2	Yes	14633.7	Early and late gametocytes
E84/21	Yes	307.6	No	No	0.0	No	0.0	Yes	165.8	pfs16 negative
E85/0	Yes	3181826.2	Yes	Yes	5.6	Yes	89.4	No	0.0	Early gametocytes only
E85/14	Yes	1716072.3	Yes	Yes	27.5	Yes	846.4	Yes	399751.8	Early and late gametocytes
E85/21	Yes	1146713.4	Yes	Yes	27.0	Yes	1654.3	Yes	375423.1	Early and late gametocytes
E89/0	Yes	7940950.2	Yes	Yes	49.2	Yes	2638.7	Yes	18752.2	Early and late gametocytes
E89/07	Yes	810479.7	Yes	Yes	316.1	No	0.0	Yes	5928659.9	Early and late gametocytes
E89/14	Yes	155395.1	Yes	Yes	36.7	No	0.0	Yes	494843.7	Early and late gametocytes
E89/21	Yes	67513.8	Yes	No	0.0	No	0.0	Yes	34632.7	Late gametocytes only
E90/0	Yes	21197671.9	Yes	Yes	944.5	Yes	429353.2	Yes	194.4	Early and late gametocytes
E90/07	Yes	85792.2	Yes	Yes	1298.6	Yes	1207.5	Yes	142418.0	Early and late gametocytes
E90/14	Yes	16687845.7	Yes	Yes	418.1	Yes	963.9	Yes	14699.6	Early and late gametocytes
E90/21	Yes	717095.8	Yes	No	0.0	No	0.0	Yes	194.9	Late gametocytes only
E92/0	Yes	4030785.6	Yes	No	0.0	Yes	5801.5	No	0.0	Early gametocytes only
E92/07	Yes	3935.6	Yes	No	0.0	No	0.0	Yes	3083.9	Late gametocytes only
E92/14	Yes	5779.4	Yes	No	0.0	Yes	1454.4	Yes	3917.1	Early and late gametocytes
E92/21	Yes	1673.5	Yes	No	0.0	Yes	24385043.1	Yes	7523.3	Early and late gametocytes

E96/0	Yes	1019993.4	Yes	No	0.0	No	0.0	Yes	6675.6	Late gametocytes only
E96/07	Yes	101498.0	Yes	No	0.0	Yes	9830.5	Yes	56989.1	Early and late gametocytes
E96/21	Yes	107921.7	No	No	0.0	No	0.0	No	0.0	pfs16 negative
E98/0	Yes	14957978.5	No	No	0.0	Yes	385.2	No	0.0	pfs16 negative
E98/07	Yes	1162.4	No	No	0.0	No	0.0	No	0.0	pfs16 negative
E98/14	Yes	1361.7	No	No	0.0	No	0.0	No	0.0	pfs16 negative
E98/21	Yes	734.6	No	No	0.0	No	0.0	No	0.0	pfs16 negative
E100/0	Yes	4897176.3	Yes	Yes	161.4	Yes	3341.1	Yes	33.2	Early and late gametocytes
E100/07	Yes	1071652.1	Yes	Yes	10553.1	Yes	30994.7	Yes	24519.8	Early and late gametocytes
E100/14	Yes	404063.6	Yes	Yes	10158.0	Yes	1138.8	Yes	363969.9	Early and late gametocytes
E102/0	Yes	35490718.8	Yes	Yes	23.7	Yes	168.0	No	0.0	Early gametocytes only
E102/14	Yes	105662.5	Yes	Yes	7.8	No	0.0	Yes	36519.3	Early and late gametocytes
E102/21	Yes	739552.1	Yes	Yes	3.3	No	0.0	Yes	4137.4	Early and late gametocytes
E103/0	Yes	101420968.8	Yes	Yes	3835.6	Yes	8141.9	Yes	2314.3	Early and late gametocytes
E103/07	Yes	181.0	No	No	0.0	Yes	2982.6	No	0.0	pfs16 negative
E103/14	Yes	79.3	Yes	Yes	49.1	Yes	711.2	No	0.0	Early gametocytes only
E103/21	Yes	165.4	No	Yes	405.6	No	0.0	Yes	1762.5	pfs16 negative
E104/0	Yes	20047294.9	Yes	Yes	199.5	Yes	3316.6	No	0.0	Early gametocytes only
E104/07	Yes	671.5	No	No	0.0	No	0.0	No	0.0	pfs16 negative
E104/14	Yes	138872.3	Yes	Yes	69.0	No	0.0	Yes	58189.8	Early and late gametocytes
E104/21	Yes	2009.7	Yes	Yes	28.7	No	0.0	Yes	11652.5	Early and late gametocytes
E105/0	Yes	943950.1	Yes	No	0.0	No	0.0	Yes	1077.6	Late gametocytes only
E105/07	Yes	2291.0	Yes	No	0.0	Yes	16718.3	No	0.0	Early gametocytes only
E105/14	Yes	2910.9	Yes	No	0.0	Yes	50075.5	Yes	62106.1	Early and late gametocytes
E105/21	Yes	2191.9	Yes	No	0.0	Yes	1116.7	Yes	110932.4	Early and late gametocytes
E106/0	Yes	9702050.8	Yes	Yes	196.3	No	0.0	Yes	1218.5	Early and late gametocytes
E106/07	Yes	349.8	Yes	Yes	3.3	No	0.0	No	0.0	Early gametocytes only
E106/14	Yes	76.5	No	No	0.0	No	0.0	No	0.0	pfs16 negative
E106/21	Yes	59.5	No	No	0.0	No	0.0	No	0.0	pfs16 negative

PID Patient ID

Total parasite	Presence of any parasite stage
Q.Total parasite	Total parasite quantity/mL of blood
Any Gametocyte	Presence of any gametocyte stage (pfs16)
Early (*pfpeg4*)	Presence of early gametocyte (*pfpeg4*)
Q.Early (*pfpeg4*)	Early gametocyte quantity (*pfpeg4*)/mL of blood
Early (*pfg27*)	Presence of early gametocyte (*pfg27*)
Q. Early (*pfg27*)	Early gametocyte quantity (*pfg27*)/mL of blood
Late (*pfs25*)	Presence of late gametocyte (*pfs25*)
Q.Late (*pfs25*)	Late gametocyte quantity (*pfs25*)/mL of blood

Coloured cells represents samples with detectable early gametocytes markers expression, though their quantities are below the limit of quantification (the lowest ivcDNA concentration that detectable in all of the experiments and falls within the log-linear relationship of the qRT-PCR assay)

Table S5: Raw data for volunteers samples

ID	Day	Tparasite.mlb	MGCTmlbl	EGCTpeg4.mlbl	EGCT27.mlbl	C_T40S
S035	7.0	15721	0.0	0.0	0.0	34.9
S035	7.5	4599	270.1	0.0	0.0	35.3
S035	8.0	42190	0.0	0.0	0.0	37.3
S035	8.1	91857	0.0	0.0	139.2	35.8
S035	8.2	126694	167.1	105.9	247.2	34.0
S035	8.5	131359	175.3	850.3	0.0	32.2
S035	8.7	157358	164.4	144.3	0.0	32.4
S035	8.8	45510	0.0	218.4	112.5	35.2
S035	9.0	7087	0.0	0.0	0.0	37.1
S035	9.3	1281	0.0	0.0	0.0	39.4
S042	7.0	14396	0.0	196.2	0.0	37.9
S042	8.0	109920	0.0	319.0	0.0	35.7
S042	8.1	289620	170.7	461.6	302.1	34.5
S042	8.2	333068	279.4	363.0	157.6	32.9
S042	8.5	227850	247.1	271.8	370.6	34.3
S042	8.7	114926	80.0	618.7	285.9	35.5
S042	8.8	68042	190.7	203.0	138.3	33.1
S042	9.0	6634	0.0	486.6	147.1	38.8
S042	9.3	836	0.0	0.0	0.0	41.3

ID Volunteer ID
Day Day of follow up
Tparasite.mlb Total parasite density/ mL of blood
MGCTmlbl Late gametocytes/ mL of blood
EGCTpeg4.mlbl Early gametocytes (pfpeg4)/mL of blood
EGCT27.mlbl Early gametocytes (pfg27)/mL of blood
C_T40S PCR cycle threshold of pf40S (endogenous control gene)

40S qRT-PCR was used to detect any DNA carryover in the purified RNA samples (reverse transcriptase-free sample), cycle threshold of pf40S was undetermined in all of the samples above indicating no DNA contamination.

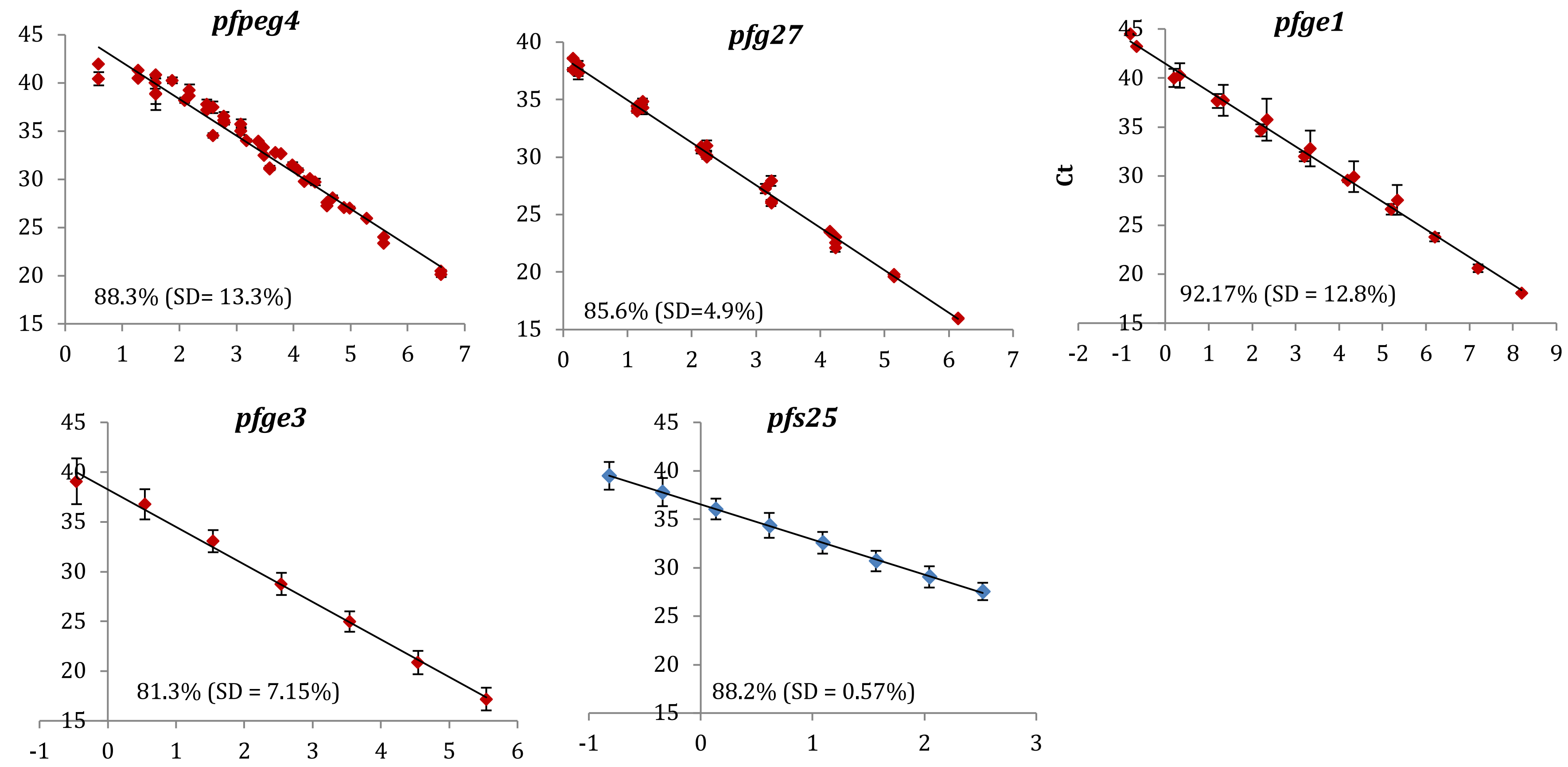

Figure S1: *pfpeg4*, *pfg27*, *pfge1*, *pfge3* and *pfs25* quantification standard curves. Standard curve generated from serially diluted ivcDNA (methods section 2.6.4). The average efficiency of the standard curves and standard deviation (SD) are given within each plot. Y axis represents the cycle threshold (C_T) at which the change in the probe fluorescence was first detectable. X axis represents log10 of the ivcDNA concentration (transcript copies/µL of ivcDNA) for *pfpeg4*, *pfg27*, *pfge1* and *pfge3* standard curves. For *pfs25* the x axis represents mature gametocytes/µL of cDNA. Error bars represent the standard deviation of the C_T replicates.

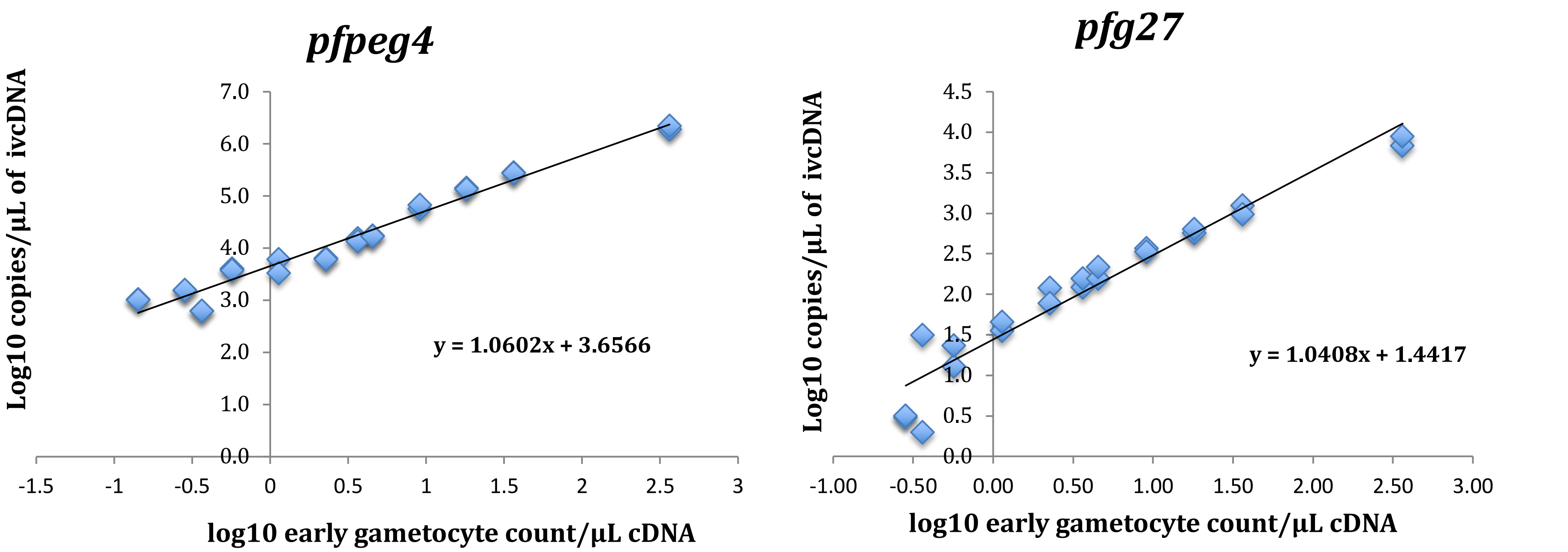

Figure S2: Conversion from transcript number to gametocytes count using *pfpeg4* and *pfg27* genes. Standard curve generated from serially diluted ivcDNA (Y axis) and purified stage II gametocytes (X axis). No conversion curves were produced for the other early gametocytes markers because it was proved that they were less specific (*pfge1* and *pfgexp5*) or lower in abundance (*pfge3*).

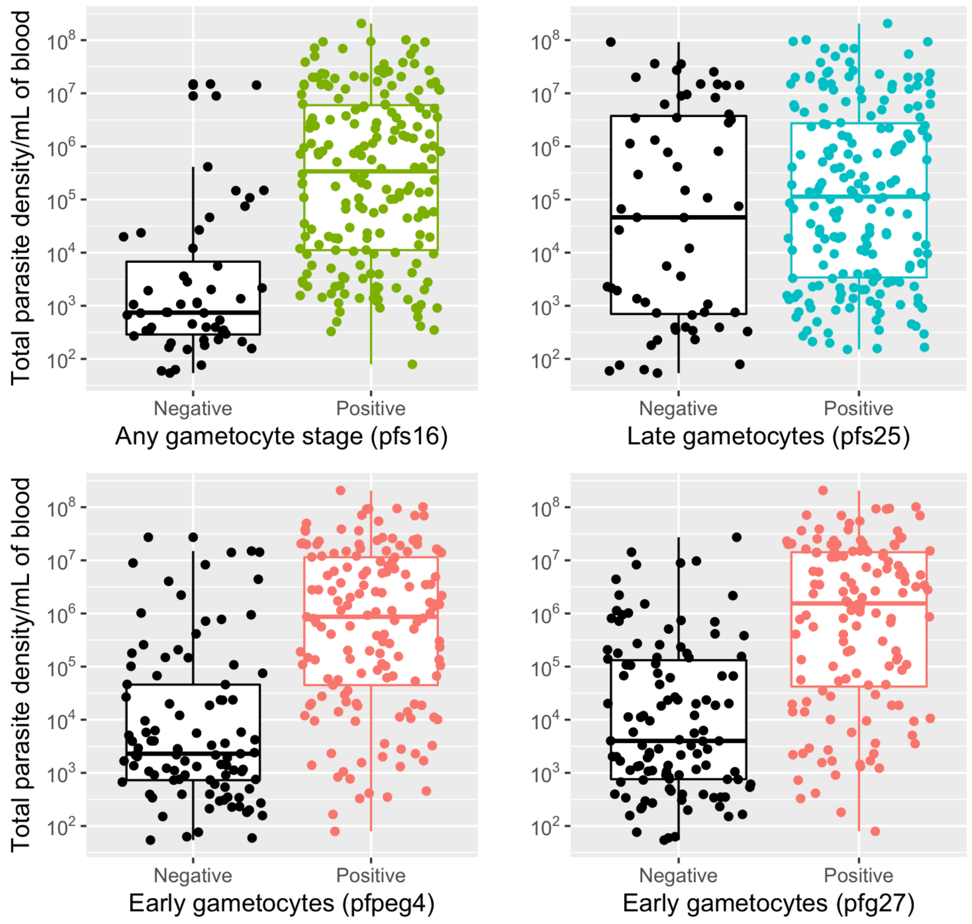

Figure S3: Detection of gametocytes stages across a wide range of parasitaemia.

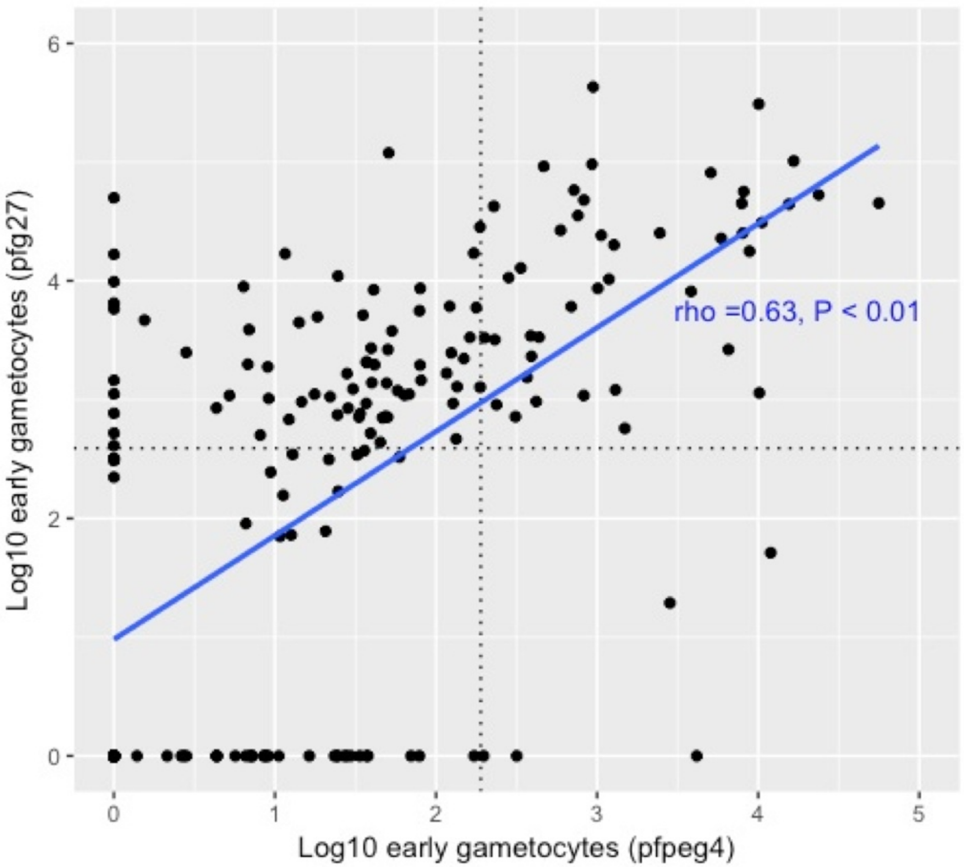

Figure S4: Correlation between log10 early gametocyte densities as detected by *pfpeg4* and *pfg27*. The vertical and horizontal dotted lines represent the limit of quantification (LoQ) for *pfpeg4* (equivalent to 190 gametocytes/ml) and *pfg27* (equivalent to 390 gametocytes/ml), respectively.
